# Occupational cognitive stimulation, socioeconomic status, and cognitive functioning in young adulthood

**DOI:** 10.1016/j.ssmph.2022.101024

**Published:** 2022-01-11

**Authors:** Rebecca C. Stebbins, Yang Claire Yang, Max Reason, Allison E. Aiello, Daniel W. Belsky, Kathleen Mullan Harris, Brenda L. Plassman

**Affiliations:** a Social, Genetic, & Developmental Psychiatry Centre, Institute of Psychiatry, Psychology, and Neuroscience, King's College London, London, UK; b Carolina Population Center, University of North Carolina-Chapel Hill, Carolina Square-Suite 210, 123 West Franklin St., Chapel Hill, NC, 27516, USA; c Department of Sociology, University of North Carolina-Chapel Hill, 155 Hamilton Hall, CB #3210, Chapel Hill, NC, 27599, USA; d Lineberger Comprehensive Cancer Center, University of North Carolina-Chapel Hill, 450 West Drive, CB #7295, Chapel Hill, NC, 27599, USA; e Department of Epidemiology, Gillings School of Global Public Health, University of North Carolina-Chapel Hill, 135 Dauer Drive, CB #7400, Chapel Hill, NC, 27599, USA; f Butler Aging Center, Mailman School of Public Health, Columbia University, 722 West 168th St., New York, NY, 10032, USA; g Department of Psychiatry & Behavioral Science, School of Medicine, Duke University, Duke University Medical Center, Box 3950, Durham, NC, 27710, USA

**Keywords:** Memory, Analytic skills, Social interaction, Occupational characteristics

## Abstract

**Background:**

Occupational characteristics are associated with late-life cognition. However, little is known about the association between occupational factors and cognition in early adulthood, especially when controlling for early life socioeconomic status (SES) and cognition in childhood. Importantly, sex may shape the impact of occupational characteristics that provide cognitive stimulation given that education, occupational status, and workplace experiences differ by sex.

**Methods:**

Using data on 12,129 participants ages 24–32 from the U.S.-based National Longitudinal Study of Adolescent to Adult Health, we investigated the association between four factors of occupational cognitive stimulation (repetition, freedom, analytic skills, and social interaction) and young-adult episodic and working memory independent of childhood and young-adult SES, using linear regression. We adjusted for confounding due to sex, race/ethnicity, age, childhood cognition, and education. We further investigated effect measure modification of this association by sex in stratified regression models.

**Results:**

Overall, 1-unit increases in both occupational analytic skills and social interaction were significantly associated with 0.101 (95%CI: 0.28, 0.173) and 0.096 (95%CI: 0.032, 0.160) SD higher memory, respectively. However, when sex-stratified, among men, a 1-unit increase on the social interaction scale was associated with 0.16 (95%CI: 0.05, 0.27) SD higher memory, while there was no association among women.

**Conclusion:**

Our results indicate that even in adulthood, activities that stimulate the mind can contribute to improved cognitive function, and the most beneficial forms of occupational stimulation are those that use analytic skills and involve social interaction (particularly among young men).

## 1 Introduction

It is well established that socioeconomic advantage across the lifecourse is positively associated with health in adulthood. Higher socioeconomic status (SES) such as that indicated by a higher education can ensure better health across life because it can be translated into greater access to health promoting resources (Link & Phelan, 1995; Phelan et al., 2010) and buffer the negative health effects of exposure to social stressors(Pearlin et al., 1981; Thoits, 2010). Prior research has generally found that both early life SES and adulthood education have independent and additive effects on cognition at older ages(Horvat et al., 2014; Landy et al., 2017; Luo & Waite, 2005; Lyu, 2015; Lyu & Burr, 2016; Marden et al., 2017; Richards & Sacker, 2003). However, some researchers have provided evidence that the effect of early-life SES on measures of cognition are completely mediated by adult educational attainment (Singh-Manoux et al., 2005; Zeki Al Hazzouri, Haan, Galea, & Aiello, 2011), and research that has assessed SES trajectories across the lifecourse has also led to conflicting results(Haan et al., 2011; Zeki Al Hazzouri, Haan, Kalbfleisch, et al., 2011).

An individual's occupation is a measure of SES that has long been considered an important predictor of health. The assumption here is that the occupation of an individual affects health indirectly through economic, cultural, or social capital accumulated as a result of this occupation, which may be invested in health-promoting resources(Fujishiro et al., 2010; Phelan et al., 2010). However, the function that occupation plays in determining *cognitive* health in adulthood is unique and may transcend its role as a measure of access to health promoting resources. Several studies have demonstrated that stimulating and complex work is associated with higher cognitive function(Nexø et al., 2016). Occupations can act as environments that either promote or inhibit cognitive stimulation through the complexity and degree of control over one's work. High-complexity jobs require that employees work through mentally stimulating tasks and engage in communication with co-workers and others while on the job. In contrast, low-complexity jobs often involve repetitive tasks that require little mental effort or social interaction(Fay & Kamps, 2006). Jobs with high control or freedom, on the other hand, enable autonomy when carrying out the tasks of the job, while low-control jobs give workers little opportunity to decide the best way to complete job-related tasks(Zacher & Frese, 2011).

High complexity jobs have been associated with a lower risk of dementia in later life but there is little research investigating whether this is manifested earlier in life with differences in cognitive function(Kröger et al., 2008). Prior research on occupation and cognition has found that occupations defined by repetitive, low-skilled tasks with low levels of control are associated with lower cognitive functioning (Gajewski et al., 2010), while individuals employed in jobs with greater mental demands and that require more complex social interactions perform better on cognitive tests(Andel et al., 2015; Karp et al., 2009; Marquié et al., 2010; Potter et al., 2008). In addition to the epidemiological evidence that mentally stimulating, high-control jobs are associated with better cognitive functioning and delayed cognitive decline, there is also evidence that these jobs are associated with both greater hippocampal volume and a slower decline in this hippocampal volume over time (Valenzuela et al., 2008), as well as higher levels of cognitive functioning controlling for hippocampal volume(Boots et al., 2015).

Importantly, gender may shape the impact of these occupational stimulating factors on cognition. Educational attainment, occupational status, and work complexity are differential by gender, where the experiences and expectations of performance can be different for men than women(Gerstorf et al., 2006; Rocca et al., 2014). Women also tend to have higher levels of social integration and support than men(Antonucci, 1994; Fuhrer & Stansfeld, 2002). Differential exposures to psychosocial stressors and social support may mediate gender differences in cognitive function and change throughout life. Furthermore, higher childhood and adult SES are associated with higher memory scores among men than women in old age (Lyu, 2015), and women benefited cognitively from social engagement more (Thomas, 2011; Zunzunegui et al., 2003) and suffered higher risk of cognitive impairment from social isolation than men(Artero et al., 2008).

The existing studies of SES or occupation and cognition come from Europe (Horvat et al., 2014; Landy et al., 2017; Singh-Manoux et al., 2005) or have focused on older individuals (i.e. ≥ 50 years).(Haan et al., 2011; Luo & Waite, 2005; Lyu, 2015; Lyu & Burr, 2016; Marden et al., 2017; Zeki Al Hazzouri et al., 2011a, 2011b). Decline in cognitive functions such as memory may begin earlier in life(Hartshorne & Germine, 2015). Data are needed that observe how job characteristics relate to poorer cognitive functioning in young adults in the US. Particularly at issue is that those with higher cognitive function in early-life select into jobs requiring stronger analytic and social skills and allowing more freedom. This is important because an observed association could be related to prior cognitive function predicting the types of occupational roles that individuals obtain. Disentangling the role of education provides another level of complexity because education could affect cognition, which then predicts occupational roles. Therefore, in order to accurately assess this relationship, studies need to control for pre-employment cognition and SES and current education, information which is often unavailable.

This study addressed these gaps by examining the association of occupational cognitive stimulation and cognitive functioning in young adulthood in a dataset that allows us to control for some of the factors that could explain selection. We examined the association of cognitive function with four domains of occupational cognitive stimulation while adjusting for adolescent cognitive function and socioeconomic status (SES) and adulthood education. We then investigated modification of these associations by sex. By utilizing a longitudinal sample of young-adult respondents with lifecourse information on SES and cognition, along with data that integrate self-reported occupational measures and direct measures of the analytic intensity and social interaction inherent to an occupation, this study aims to advance our knowledge about the pathways between education, occupation, and cognition across the lifecourse.

## 2 Methods

### 2.1 Study population

We used data from Waves I and IV of the National Longitudinal Study of Adolescent to Adult Health (Add Health)(Harris et al., 2019). Add Health is a U.S.-representative sample of middle- or high-schoolers during the 1994–1995 school year who have been followed with four additional interviews (Waves II-V) collecting information on health, health behaviors, well-being, socioeconomic security, and education. Data for Wave I were collected when respondents were aged 12–19 years through in-school and in-home interviews and included a measure of verbal ability. Parent interviews were also conducted to gain information on the socioeconomic status and household situation of the child during adolescence. The Wave IV survey occurred in 2008, and included cognitive measures as well as self-reported current or most recent occupation. Of the 15,701 respondents who were interviewed at Wave IV, our analysis included 12,129 who had proper sampling weights, had proper occupational codes and were not in the military, or were not missing covariate or outcome data.

### 2.2 Measures

#### 2.2.1 Young-adult cognitive function

In Add Health Wave IV, episodic memory was assessed with immediate word recall, delayed word recall, and working memory was assessed with digits backwards recall. In the word recall tasks, respondents were first read a list of 15 words and then asked to immediately recall as many as they could remember (*immediate recall)*. The number of words correctly recalled after a 5-min delay formed the *delayed word recall* score. For the *digits backward recall* task, respondents were read a set of numbers and asked to recall them in reverse order. The task was repeated up to seven times, each time with a longer series of numbers. The task was ended after the seventh number series or after the participant could not complete a series within two attempts. After standardizing the scores on all three measures to a mean of zero and a standard deviation of one, the three measures were summed. This summed score was then re-standardized to a mean of zero and SD of one.

#### 2.2.2 Adolescent verbal cognitive ability

We used scores from the Add Health Picture Vocabulary Tests (AHPVT) administered to respondents during the Wave I survey. An abridged, computerized version of the Peabody Picture Vocabulary Test-Revised (PPVT), the AHPVT tested the overall vocabulary knowledge of the adolescents by requiring respondents to match illustrations to words that best fit together and is highly correlated with the full Peabody Picture Vocabulary test (PPVT)(Halpern et al., 2000; Harris et al., 2019). The AHPVT scores are age-standardized and scaled from 14 to 146.

##### 2.2.2.1 Occupational cognitive stimulation

Occupational cognitive stimulation was defined using survey data collected from Add Health participants about their jobs at the Wave IV assessment and data from the Occupational Information Network (O*NET) (Development, 2018) linked to occupations using SOC2000 codes.

Respondents reported on Likert scales about *repetitive work* (how repetitive the tasks required for their job were, i.e. non-complex) and *job-task freedom* (how much control they had to make decisions about the tasks they performed at their work). Response options were “None or almost none of the time” to “All or almost all of the time”, coded 0–3 for analysis.

O*NET classifications of occupations were used to define *analytic skills of occupation* and *social interaction of occupation,* which measure job complexity. These measurements were derived from items that coded the importance of different daily tasks required for the occupation. For each item on the O*NET occupational survey, respondents reported the importance of a given task on a five-point Likert scale from 1 to 5, in which 1 was “Not important” and 5 was “Extremely important”. Responses across all respondents within an occupation were averaged, leading to a single importance score for each item from 1 to 5. Using these items, O*NET has created a content model in which individual items are conceptually grouped together under larger constructs.

Using a content model provided by O*NET, mean scores were created from individual job-task items within conceptual “constructs” that defined the broader domains of skills defined by the individual tasks. Those constructs that best measured occupational analytic demands and frequency of social interaction were then re-averaged within each occupation. Within the current study, the variable *Analytic Skills of Occupation* was created using the constructs of “Process”, “Complex Problem Solving”, and “Practical Intelligence”; for *Social Interaction of Occupation*, the constructs utilized were “Interpersonal Interaction” and “Communicating and Interacting”. A list of each individual O*NET item used in the creation of these constructs can be found in Table 1. Within each occupational category, the mean of all the items within a construct was taken and used as a value for the construct. For the final variables used in this study, the mean of all relevant constructs was taken, leading to a final value of analytic skills and social interaction necessary for each occupation. For these variables, higher values correspond to greater use of each of these skills.

**Table 1:** Description of O*NET variables used in the present study.

Occupation Variable	O*NET Construct	Individual O*NET Item
Analytic Skills	Process	Critical Thinking • Using logic and reasoning to identify the strengths and weaknesses of alternative solutions, conclusions or approaches to problems.
		Active Learning • Understanding the implications of new information for both current and future problem-solving and decision-making.
		Learning Strategies • Selecting and using training/instructional methods and procedures appropriate for the situation when learning or teaching new things.
		Monitoring • Identifying complex problems and reviewing related information to develop and evaluate options and implement solutions.
	Complex Problem Solving	Complex Problem Solving • Identifying complex problems and reviewing related information to develop and evaluate options and implement solutions.
	Practical Intelligence	Innovation • Job requires creativity and alternative thinking to develop new ideas for and answers to work-related problems.
		Analytical Thinking • Job requires analyzing information and using logic to address work-related issues and problems.
Social Interaction	Interpersonal Orientation	Cooperation • Job requires being pleasant with others on the job and displaying a good-natured, cooperative attitude.
		Concern for Others • Job requires being sensitive to others' needs and feelings and being understanding and helpful on the job.
		Social Orientation • Job requires preferring to work with others rather than alone, and being personally connected with others on the job.
	Communicating and Interacting	Interpreting the Meaning of Information for Others • Translating or explaining what information means and how it can be used.
		Communicating with Supervisors, Peers, or Subordinates • Providing information to supervisors, co-workers, and subordinates by telephone, in written form, e-mail, or in person.
		Communicating with Persons Outside Organization • Communicating with people outside the organization, representing the organization to customers, the public, government, and other external sources. This information can be exchanged in person, in writing, or by telephone or e-mail.
		Establishing and Maintaining Interpersonal Relationships • Developing constructive and cooperative working relationships with others, and maintaining them over time.
		Assisting and Caring for Others • Providing personal assistance, medical attention, emotional support, or other personal care to others such as coworkers, customers, or patients.
		Selling or Influencing Others • Convincing others to buy merchandise/goods or to otherwise change their minds or actions.
		Resolving Conflicts and Negotiating with Others • Handling complaints, settling disputes, and resolving grievances and conflicts, or otherwise negotiating with others.
		Performing for or Working Directly with the Public • Performing for people or dealing directly with the public. This includes serving customers in restaurants and stores, and receiving clients or guests.

#### 2.2.3 Early-life socioeconomic status and adult education

Early-life SES was measured using a Social Origins Score originally created by Belsky et al., in which Wave I information on parental education, parental occupation, household income, and household receipt of public assistance were combined and standardized within the Add Health (Belsky et al., 2018). Adult educational attainment was categorized as less than a high school diploma, high school diploma, some college, or a college diploma and higher. We also adjusted for adult income measured by the log-transformed reported individual earnings of the respondent in the last full calendar year in a supplemental model.

#### 2.2.4 Covariates

Sociodemographic characteristics including race and ethnicity, sex, and age were collected at Wave IV. Race and ethnicity were categorized as non-Hispanic White, non-Hispanic Black, Hispanic, and “other”, which combined all other racial and ethnic groups due to small numbers, and sex was self-reported as either male or female.

### 2.3 Statistical analyses

All analyses were conducted in SAS 9.4 (SAS Institute, Cary, NC). Descriptive statistics were used to characterize the study population. To estimate the association between occupational cognitive stimulation and young adulthood episodic and working memory, we used linear regression models with all four components of cognitive stimulation as independent variables. The final, minimally-sufficient adjustment set was determined *a priori*. Model 1 is unadjusted save for adolescent cognitive ability, while Model 2 adds demographic characteristics, and Model 3 adds the lifecourse socioeconomic characteristics of childhood SES and adult educational attainment. We further evaluated potential effect measure modification by biological sex with exposure*sex interaction terms to test for statistical significance followed by stratified models to estimate measures of association. In all models, we used sample weights and cluster robust standard errors to account for Add Health's complex survey design. Sensitivity analyses were conducted to adjust for participant income.

## 3 Results

### 3.1 Sample characteristics

The 12,129 participants who met eligibility criteria were 49.4% female and a mean of 28 (SD: 1.8) years old. The majority of the study population primarily identified as non-Hispanic White (70.7%), with 14.4% identifying as non-Hispanic Black. This distribution was similar for men and women. The most prevalent educational attainment was “some college”, with 43% of participants reporting this achievement and 32% reporting completing a college degree or higher education. More women than men received a college degree (36% and 28%, respectively). However, mean personal income was significantly higher among men ($40,669 (SD: $38,962)) compared to women ($28,197 (SD: $31,598)). Mean standardized memory was higher among women (0.10 (SD: 0.99)) than men (−0.12 (SD: 1.01)). The complete demographic and social characteristics of the study population are shown in Table 2. Supplemental Table 1 shows the age-, sex-, and race-adjusted associations of socioeconomic and childhood cognitive variables with the four occupational cognitive stimulation variables.

**Table 2:** Weighted Sociodemographic and Memory Characteristics of Eligible Add Health Participants, n = 12,129.

	Overall		Women		Men		Memory Score	
	N	%	N	%	N	%	Mean	SD
Age, mean (SD)	28.28	1.82	28.18	1.80	28.38	1.84		
Sex								
*Female*	6469	49.44					0.10	0.99
*Male*	5660	50.56					−0.12	1.01
Race/Ethnicity								
*non-Hispanic White*	7037	70.67	3710	70.75	3327	70.59	0.12	0.98
*non-Hispanic Black*	2458	14.40	1397	14.64	1061	14.18	−0.41	0.97
*Hispanic*	1764	10.62	933	10.49	831	10.74	−0.29	1.04
*Other*	870	4.31	429	4.12	441	4.49	−0.01	0.98
Childhood SES, mean (SD)	0.04	1.327	0.03	1.32	0.05	1.33		
Education								
*< High School*	827	8.16	338	6.42	489	9.87	−0.60	0.94
*High School Diploma*	1862	16.71	834	13.12	1028	20.22	−0.36	0.97
*Some College*	5341	43.16	2852	44.51	2489	41.84	−0.04	0.96
*College +*	4099	31.96	2445	35.95	1654	28.07	0.36	0.96

Personal Earnings ($), mean (SD)	34503	36035	28197	31598	40669	38926		

Occupational Cognitive Stimulation, mean (SD)								
*Repetitive Work*	1.89	0.92	1.96	0.91	1.82	0.93		
*Freedom at Work*	1.91	0.95	1.82	0.94	1.99	0.95		
*Analytic Skills*	3.30	0.38	3.32	0.38	3.28	0.38		
*Social Interaction*	3.60	0.37	3.72	0.34	3.49	0.37		

Adolescent Verbal Cognitive ability, mean (SD)	102.46	13.78	101.93	13.85	102.97	13.70		

### 3.2 Occupational cognitive stimulation and cognition

When only adjusting for adolescent verbal cognitive ability (Model 1), occupational analytic skills, repetitive work, and social interaction at work were all significantly associated with memory scores. These associations were attenuated in models adjusted for sociodemographic characteristics and education (Model 3), where we found that 1-unit increases in occupational characteristics were significantly associated with 0.10 ((95%CI: 0.03, 0.18); analytic skills)) and 0.10 ((95%CI: 0.03, 0.16); social interaction)) SD higher memory. Full results can be found in Table 3. We found no meaningful changes in the association when further adjusting for participant earnings in sensitivity analysis (Supplemental Table 2).

**Table 3:** Estimates and 95% Confidence Intervals from Linear Regression of Memory (z-score) in Add Health, n = 12,129.

	Model 1				Model 2				Model 3			
	estimate	95% CI			estimate	95% CI			estimate	95% CI		
Intercept	−3.91	−4.30	−3.51	***	−2.62	−3.27	−1.96	***	−1.98	−2.59	−1.38	***
Occupational Cognitive Stimulation												
Freedom	0.01	−0.02	0.03		0.02	−0.01	0.04		0.02	−0.01	0.04	
Repetitive Work	−0.04	−0.06	−0.01	**	−0.04	−0.07	−0.01	**	−0.02	−0.04	0.01	
Analytic Skills	0.23	0.15	0.30	***	0.24	0.17	0.31	***	0.10	0.03	0.18	**
Social Interaction	0.23	0.17	0.29	***	0.14	0.07	0.20	**	0.10	0.03	0.16	**
Adolescent Verbal Cog Ability	0.02	0.02	0.03	***	0.02	0.02	0.02	***	0.02	0.02	0.02	***
Age					−0.03	−0.05	−0.01	**	−0.03	−0.05	−0.01	**
Female					0.20	0.15	0.26	***	0.18	0.13	0.23	***
Race/Ethnicity												
non-Hispanic Black					−0.22	−0.30	−0.14	***	−0.23	−0.31	−0.15	***
Hispanic					−0.15	−0.26	−0.03	*	−0.13	−0.24	−0.01	*
Other					−0.03	−0.12	0.07		−0.07	−0.16	0.03	
non-Hispanic White					ref.				ref.			
Childhood SES									0.02	0.00	0.05	
Educational Attainment												
High School Diploma									0.16	0.04	0.28	**
Some College									0.30	0.20	0.39	***
College +									0.49	0.38	0.59	***
< High School									ref.			

*p < 0.05, **p < 0.01, ***p < 0.0001.

### 3.3 Modification by sex

Overall, only the association between social interaction at work and memory was modified by sex. The magnitude of the association or lack-thereof with analytic skills, freedom, and repetitive work and memory were not meaningfully different in men or women. In sex-stratified models, social interaction was only significantly associated with memory among men and not among women. Among men, a 1-unit increase on the social interaction scale was associated with 0.17 (95%CI: 0.06, 0.28) SD higher memory, while there was virtually no association with social interaction and memory among women (β = 0.01 (95%CI: −0.09, 0.12)). For all other occupational characteristics, the point estimates were not meaningfully different and the confidence intervals included each other's estimates. Full results for the sex-stratification are presented in Table 4.

**Table 4:** Sex-stratified Estimates and 95% Confidence Intervals from Linear Regression of Memory in Add Health, n = 12,129.

	Men						Women						Interaction p-value
	Model 1			Model 3			Model 1			Model 3			
	estimate	95% CI		estimate	95% CI		estimate	95% CI		estimate	95% CI		
Occupational Cognitive Stimulation													
Freedom	0.02	−0.02	0.07	0.03	−0.01	0.07	0.01	−0.02	0.04	0.00	−0.04	0.03	0.59
Repetitive Work	−0.05	−0.08	−0.02	−0.03	−0.06	0.00	−0.04	−0.08	0.00	0.00	−0.04	0.04	0.40
Analytic Skills	0.23	0.13	0.32	0.09	−0.01	0.18	0.24	0.15	0.33	0.12	0.03	0.21	0.84
Social Interaction	0.21	0.11	0.31	0.17	0.06	0.28	0.04	−0.07	0.15	0.01	−0.09	0.12	0.04

## 4 Discussion

In this study, we examined the associations between occupational cognitive stimulation and memory in young adulthood, a developmental period for which research on the social and occupational determinants of cognitive functioning is scant. Our results show that overall, more analytical skills and greater social interaction characteristics of one's occupation are associated with better memory in young adulthood, independent of both adolescent socioeconomic status and pre-employment cognitive performance as well as adulthood education. The relationship between analytic skills and memory were consistent for both sexes, but we found that the magnitude of the association of occupational social interaction with memory was modified by sex: among men, a 1-unit increase in occupational social interaction was associated with 0.17 SD (95%CI: 0.06, 0.28) increase in memory score, while there was virtually no association between occupational social interaction and memory among women. We found no associations between repetitive work or freedom at work and memory when other occupational characteristics were adjusted.

Our findings differed from some previous studies regarding specific aspects of occupations that were associated with memory in adulthood, particularly occupational social interaction. Furthermore, while the modification of the association between occupational social interaction and memory by sex was anticipated and consistent with much of the previous literature, our results were inconsistent in that previous studies have found that women benefit more from social engagement than men (Thomas, 2011; Zunzunegui et al., 2003), while our study found the opposite. However, the prior research was among individuals in old age, rather than early in the lifecourse. Given that our study was among young adults, who are often more socially-integrated than older adults (who may have social lives centered more on family), workplace social interaction may provide different benefits. Men may benefit more than women from social interaction at work, in this young adult population. However, this finding may also reflect that men and woman have different specific jobs that score high on this occupational characteristic, and thus require different types of social interaction. Specifically, women may rank more highly on social interaction scale due to having jobs that require a high level of concern and care for others (e.g. nursing, teaching) while men may rank highly on the scale from jobs requiring selling or influencing others (e.g. salesman, lawyer). These different skills and occupations that are ultimately ranked the same on the social interaction scale due to different on-the-job tasks may mean that the benefits for cognition differ for men and women.

Surprisingly, our study found no association between either self-reported task repetition (a relationship hypothesized to be negative) or self-reported job task freedom (a relationship hypothesized to be positive) and memory, as has been found in previous research(Andel et al., 2015; Karp et al., 2009; Kröger et al., 2008; Marquié et al., 2010; Potter et al., 2008). This may indicate that the negative effect of repetitive or low control jobs on cognitive functioning does not come to fruition until later in the lifecourse. Research that has found evidence of the relationship between cognition and these occupational factors has largely utilized samples of individuals later in their career or post-retirement. The possible long-term effect of repetitive work or low job control on cognition may not be evident in this sample due to their younger age.

Our analysis contributed new knowledge about the life-course process linking SES and occupation to memory in multiple ways: first, the use of prospective measures of life-course SES, including a measure of SES in adolescence based on information supplied directly by the parents of the survey respondents and measures of education and income in young adulthood. Second, the linkage of survey data with validated extant data on occupational characteristics allowed for the empirical test of occupational cognitive stimulation as an independent dimension of young-adult SES that can influence young-adult memory. This has moved us one step forward to understanding potential mechanisms by which conventional measures of SES may enhance memory and cognitive functioning in general. Third, we were able to adjust for adolescent intelligence, and finally, our research also adds to the existing literature by integrating occupation as a key measure of SES in influencing cognitive function.

However, our study has several limitations. First, the AHPVT does not capture the same cognitive constructs as the cognitive tests used to measure respondents’ working & episodic memory in young adulthood so these measures cannot be considered repeated tests of cognition. Therefore, we may not be adequately controlling for baseline intellect and its role in occupation-selection. However, the PPVT has been found to be an acceptable proxy for more advanced tests of intelligence in childhood.(Hodapp & Gerken, 1999). Importantly, our measure of cognition, memory, is limited to only one domain of cognitive function, as Add Health did not collect measures of other cognitive domains. This may partially explain why our findings differed from previous work using measures of cognitive functioning in different cognitive domains. Second, respondents in the Add Health sample are likely to still be transitioning through the workforce due to their young ages at Wave IV (late 20s). As a result, occupations captured here may be temporary as individuals experience upward job mobility. It is also important to note that our discussion of gendered experiences in the background is assessed with measurements of biological sex, as that is what is available in our data. However, the theoretical differences posited would be due to the social construction of gender, and the experiences of those presenting as female, rather than any inherent biological differences between men and women. Finally, there is potentially bias in our results due to measurement error in job characteristic variables, unmeasured confounding, and insufficient adjustment for baseline intellect.

In conclusion, the findings from this study suggest that cognitively stimulating occupational roles can contribute to improved cognitive function. Our results indicate that the most beneficial forms of cognitive stimulation are those which come from the use of analytic skills and social interaction, particularly for young adult men. Our study also suggests that promoting cognitive stimulation beyond the workplace could potentially counter the disadvantages due to lower SES. Future work should investigate how occupation, SES, and cognitive ability across the lifecourse are associated with not only young-adult cognition, but also how these factors influence trajectories of cognition across the middle part of the lifecourse. Further research that parses out whether long-standing gender disparities in occupation opportunities in the work place may limit benefits to cognition from occupationally stimulating jobs, is warranted.

## Author contributions

YC Yang: Designed study, directed statistical analysis, edited manuscript.

M Reason: Conducted initial analysis and drafted manuscript.

RC Stebbins: Conducted analysis and drafted manuscript.

KM Harris: Consulted on study design and provided statistical analysis and manuscript feedback.

DW Belsky: Consulted on study design and provided statistical analysis and manuscript feedback.

AE Aiello: Consulted on study design, edited the manuscript, provided input on the statistical analysis and gave manuscript feedback.

BL Plassman: Consulted on study design and provided manuscript feedback.

## Ethical statement

Add Health was approved by the UNC-Chapel Hill Institutional Review Board, and there is no identifying information available in this article.

## Declaration of competing interest

None declared.
