# Supplementary material for: Occupational cognitive stimulation, socioeconomic status, and cognitive functioning in young adulthood
Source: SSM Popul Health. 2022 Jan 11;17:101024. doi: 10.1016/j.ssmph.2022.101024 (PMC8762043; doi:10.1016/j.ssmph.2022.101024)
Supplement: Multimedia component 1 [file mmc1.pdf]

O*NET Data

O*NET is a data collection effort run by the U.S. Department of Labor and the National Center for O*NET Development (National Center for O*NET Development 2018) in which businesses around the United States were sampled and workers within these businesses were randomly selected. Those sampled were administered surveys inquiring about the importance of myriad tasks, abilities, and skills necessary in order to fulfill the requirements of their occupation. By using crosswalks available from the Bureau of Labor Statistics (Bureau of Labor Statistics 2019) and O*NET (O*NET Resource Center 2019) it was possible to link data from O*NET to the reported occupations of the Add Health respondents, as both O*NET and Add Health used SOC codes to classify occupations. In some instances, O*NET variables were not available at the finest grain of occupational classification for all respondents in Add Health. Those respondents without linkable SOC codes were assigned the value of the modal occupation (among those Add Health respondents *with* linkable SOC codes) within the next highest order of occupational classification, as individual SOC codes are nested within more general occupational categories.

Table S1. Age-, Sex-, and Race-adjusted Associations with Occupational Cognitive Characteristics

	Freedom		Repetition		Social Skills		Analytic Skills	
	mean	SE	mean	SE	mean	SE	mean	SE
Peabody Vocabulary Test	0.003	0.001	-0.012	0.001	0.003	0.000	0.006	0.000
Childhood SES	0.062	0.011	-0.138	0.011	0.041	0.004	0.084	0.004
Education								
< High School	ref.		ref.		ref.		ref.	
High School Diploma	0.082	0.054	-0.120	0.055	0.042	0.016	0.069	0.019
Some College	0.156	0.048	-0.283	0.051	0.163	0.016	0.210	0.019
College +	0.325	0.050	-0.756	0.055	0.262	0.017	0.501	0.019
Personal income	0.057	0.004	-0.046	0.005	0.006	0.002	0.031	0.002

Table S2. Income-Adjusted Estimates and 95% Confidence Intervals from Linear Regression of Working Memory in Add Health, n = 12,129

	Model 1			
	estimate	95% CI		
Intercept	-2.02	-2.62	-1.42	***
Occupational Cognitive Stimulation				
Freedom	0.01	-0.01	0.04	
Repetitive Work	-0.01	-0.04	0.01	
Analytic Skills	0.10	0.02	0.17	*
Social Interaction	0.10	0.03	0.17	**
Educational Attainment				
High School Diploma	0.16	0.04	0.27	**
Some College	0.29	0.20	0.39	***
College +	0.48	0.37	0.59	***
< High School	ref.			
Childhood SES	0.02	0.00	0.05	
Age	-0.03	-0.05	-0.01	**
Female	0.18	0.13	0.23	***
Race/Ethnicity				
non-Hispanic Black	-0.23	-0.31	-0.15	***
Hispanic	-0.13	-0.24	-0.01	*
Other	-0.07	-0.16	0.03	
non-Hispanic White	ref.			
Adolescent Verbal Cog Ability	0.02	0.02	0.02	***
log(Income)	0.01	0.00	0.01	
